# Antibacterial Effect of *Copaifera duckei* Dwyer Oleoresin and Its Main Diterpenes against Oral Pathogens and Their Cytotoxic Effect

**DOI:** 10.3389/fmicb.2018.00201

**Published:** 2018-02-21

**Authors:** Fariza Abrão, Jessica A. Alves, Gessica Andrade, Pollyanna F. de Oliveira, Sérgio R. Ambrósio, Rodrigo C. S. Veneziani, Denise C. Tavares, Jairo K. Bastos, Carlos H. G. Martins

**Affiliations:** ^1^Research Laboratory of Applied Microbiology, University of Franca, São Paulo, Brazil; ^2^Laboratory of Mutagenesis, University of Franca, São Paulo, Brazil; ^3^Nucleus of Research in Sciences and Technology, University of Franca, São Paulo, Brazil; ^4^School of Pharmaceutical Sciences of Ribeirão Preto, University of São Paulo, São Paulo, Brazil

**Keywords:** endodontic infections, dental caries, antibacterial activity, cytotoxic assay, *Copaifera duckei*

## Abstract

This study evaluates the antibacterial activity of the *Copaifera duckei* Dwyer oleoresin and two isolated compounds [eperu-8(20)-15,18-dioic acid and polyalthic acid] against bacteria involved in primary endodontic infections and dental caries and assesses the cytotoxic effect of these substances against a normal cell line. MIC and MBC assays pointed out the most promising metabolites for further studies on bactericidal kinetics, antibiofilm activity, and synergistic antibacterial action. The oleoresin and polyalthic acid but not eperu-8(20)-15,18-dioic provided encouraging MIC and MBC results at concentrations lower than 100 μg mL^−1^. The oleoresin and polyalthic acid activities depended on the evaluated strain. A bactericidal effect on *Lactobacillus casei* (ATCC 11578 and clinical isolate) emerged before 8 h of incubation. For all the tested bacteria, the oleoresin and polyalthic acid inhibited biofilm formation by at least 50%. The oleoresin and polyalthic acid gave the best activity against *Actinomyces naeslundii* (ATCC 19039) and *L. casei* (ATCC 11578), respectively. The synergistic assays combining the oleoresin or polyalthic acid with chlorhexidine did not afford interesting results. We examined the cytotoxicity of *C. duckei* oleoresin, eperu-8(20)-15,18-dioic acid, and polyalthic acid against Chinese hamster lung fibroblasts. The oleoresin and polyalthic acid were cytotoxic at concentrations above 78.1 μg mL^−1^, whereas eperu-8(20)-15,18-dioic displayed cytotoxicity at concentrations above 312.5 μg mL^−1^. In conclusion, the oleoresin and polyalthic acid are potential sources of antibacterial agents against bacteria involved in primary endodontic infections and dental caries in both the sessile and the planktonic modes at concentrations that do not cause cytotoxicity.

## Introduction

The oral bacterial microbiome encompasses ~700 commonly occurring phylotypes, about half of which can be present at any time in any individual. Oral bacteria are inseparably intertwined with diseases, such as gingivitis, periodontal diseases, endodontic infections, and dental caries, which will impact every human at some point in their lives (Palmer, 2013).

Dental caries is one of the most common biofilm-dependent oral diseases among humans (Bowen, 2002). Colonization of the tooth surface by cariogenic microorganisms, like *Streptococcus mutans, Streptococcus sobrinus*, and *Lactobacillus* spp., can destroy the tooth structure (Gross et al., 2012). *S. mutans* has been implicated as the primary etiological agent of dental caries and plays a decisive role in dental plaque formation, known as biofilm, and in dental caries development (Hamada et al., 1984; Kuramitsu and Trapa, 1984; Loesche, 1986; Rozen et al., 2001; Banas, 2004). The key to preventing such diseases is to control these cariogenic bacteria effectively. However, eliminating bacteria is a difficult task because biofilm may emerge, which enhances bacterial resistance to antimicrobial agents (Watnick and Kolter, 2000; Ding et al., 2014). Endodontic infections have a polymicrobial nature, with obligate anaerobic bacteria conspicuously dominating the microbiota in primary infections (Narayanan and Vaishnavi, 2010). Microorganisms and their products play an essential part in the development of pulp and periapical diseases and account for endodontic treatment failure (Guerreiro-Tanomaru et al., 2015). Chemomechanical preparation of the infected root canal with antimicrobial agents, followed by obturation and coronal restoration, provides a favorable outcome (Narayanan and Vaishnavi, 2010). Nevertheless, root canal treatment sometimes fails due to persistent or secondary intraradicular infection (Siqueira, 2001; Nair, 2006; Narayanan and Vaishnavi, 2010). Although chlorhexidine is usually employed as an active ingredient in mouthwash to inhibit or diminish oral bacteria, adverse reactions including bitter taste and tooth staining have limited its clinical application. Therefore, the search for alternative antibacterial agents without or with few side effects is urgent (Peng et al., 2013).

Brazil is a continental country that is recognized for housing one of the greatest plant diversities in the world. In each Brazilian region, the population uses plants according to their cultural traditions and to the types of vegetation growing therein (Brandão et al., 2013). Plants continue to be an important source of new bioactive substances, and the economic interest in prospecting them for drug discovery remains high. At least 25% of all modern medicines are estimated to derive from medicinal plants either directly or indirectly (Newman and Cragg, 2012). The oleoresin obtained by tapping the trunk of trees belonging to the *Copaifera* genus is widely used in Brazilian folk medicine under the name “oleo de copaiba” (copaiba balsam), which acts mainly as a healing, antiseptic, and anti-inflammatory agent (Cascon and Gilbert, 2000; Veiga and Pinto, 2002). The *Copaifera duckei* Dwyer oleoresin exhibits biological activities such as antiproliferative, antimutagenic, embryotoxic, anti-inflammatory, and analgesic actions (Castro-e-Silva et al., 2004; Carvalho et al., 2005; Maistro et al., 2005; Lima et al., 2011; Leandro et al., 2012). Recently, Borges et al. (2016) evaluated the *in vitro* schistosomicidal effects of the *C. duckei* oleoresin and its major secondary metabolite, (–)-polyalthic acid, to demonstrate that these substances are active against *Schistosoma mansoni* and may be employed for further investigations into compounds that can combat this parasite. Santos et al. (2013) assessed the antibacterial activity of the *C. duckei* oleoresin against bacteria of clinical and food interest, to verify that the oleoresin showed good activity against Gram-positive bacteria and acted on the bacterial cell wall of *Bacillus cereus*, affecting the cell-division process. The authors suggested that the oleoresin has a potential antibacterial effect.

This study examines the antibacterial activity of the *C. duckei* Dwyer oleoresin and its secondary metabolites against bacteria involved in primary endodontic infections and dental caries in both the planktonic mode and the sessile mode.

## Materials and methods

### Plant material and pure compounds

Authentic oleoresin from *C. duckei* Dwyer was collected in Belém, Pará (S01°06.933′O48°19.781′) by Jonas J. M. da Silva in September 2012 with the authorization of the Brazilian Ministry of Environment (protocol number: 35143-1). *C. duckei* was identified by Silvana Tavares Rodrigues from Embrapa, Belém, Pará, and the voucher specimen was deposited at the Embrapa Herbarium under number NID:96/2012. Pure (–)-polyalthic acid **(1 - PA)** and eperu-8(20)-15,18-dioic acid (**2**) were obtained according to the methodology reported by our research group (Borges et al., 2016).

### Bacterial strains and antimicrobial assays

The Minimum Inhibitory Concentration (MIC; the lowest concentration of the test compound that is capable of inhibiting microorganism growth) and the Minimum Bactericidal Concentration (MBC; defined as the lowest concentration of the test compound at which no bacterial growth occurs) of the oleoresin and the pure metabolites were determined in triplicate; the microdilution broth method in 96-well microplates was employed. Standard strains acquired from the American Type Culture Collection and clinical isolates that represent cariogenic infections were used: *Streptococcus salivarius* (ATCC 25975 and clinical isolate), *S. sobrinus* (ATCC 33478), *S. mutans* (ATCC 25275), *Streptococcus mitis* (ATCC 49456), *Streptococcus sanguinis* (ATCC 10556 and clinical isolate), *Lactobacillus casei* (ATCC 11578 and clinical isolate), and *Enterococcus faecalis* (ATCC 4082 and clinical isolate). In addition, clinical isolates and strains that best represent endodontic infections and which were acquired from the American Type Culture Collection were employed: *Porphyromonas gingivalis* (ATCC 33277 and clinical isolate), *Prevotella nigrescens* (ATCC 33563), *Fusobacterium nucleatum* (ATCC 25586 and clinical isolate), *Bacteroides fragilis* (ATCC 25285), *Actinomyces naeslundii* (ATCC 19039 and clinical isolate), *Prevotella buccae* (clinical isolate), *Aggregatibacter actinomycetemcomitans* (ATCC 43717), *Peptostreptococcus micros* (clinical isolate), *Actinomyces viscosus* (clinical isolate), *Prevotella intermedia* (clinical isolate), and *Peptostreptococcus anaerobius* (ATCC 27337). All the clinical isolates used here were provided by Brenda Paula Gomes from Faculdade de Odontologia de Piracicaba (UNICAMP). The strains were maintained at the culture collection of the Laboratory of Research in Applied Microbiology (LaPeMA/UNIFRAN) under cryopreservation at −80°C.

The following culture media were used for the cariogenic strains: Tryptic Soy Broth—TSB (Difco, Kansas City, MO, USA) and Tryptic Soy Agar—TSA (Difco) mixed with sheep blood (5%) (Nassar et al., 2012; Krzyściak et al., 2017). The culture media employed for the representative strains of endodontic infections were Schadler broth or Schadler agar (Difco), both supplemented with hemin (5.0 μg mL^−1^, Sigma, St. Louis, MO, USA), vitamin K1 (10 μg mL^−1^, Sigma), and sheep blood (5%, Bio Boa Vista, Valinhos, SP, Brazil), as recommended by CLSI (2007). Samples were dissolved in dimethyl sulfoxide (DMSO) 1.0 mg mL^−1^ and diluted in the desired broth. The concentrations ranged from 0.195 to 400 μg mL^−1^. The final DMSO content was 5% (v/v), and this solution was used as negative control. Chlorhexidine dihydrochloride (CDH) and metronidazole (Sigma) were used as positive controls for aerobic/anaerobic facultative and anaerobic bacteria, respectively. The inoculum was adjusted for each organism, to yield a cell concentration of 5 × 10^5^ colony forming units (CFU) mL^−1^ for the aerobic and anaerobic facultative strains and 5 × 10^6^ CFU mL^−1^ for the anaerobic strains according to a previous standardization by the Clinical Laboratory Standards Institute (CLSI, 2007, 2009). The anaerobic strains were incubated in an anaerobic chamber (Don Whitley Scientific, Bradford, UK) for 72 h, under atmosphere containing 5–10% H_2_, 10% CO_2_, and 80–85% N_2_. The anaerobic facultative strains were incubated in a microaerophilic jar system for 24 h, except for the *E. faecalis* (ATCC and clinical isolate) and *S. salivarius* (ATCC and clinical isolate) strains, which were incubated aerobically at 37°C for 24 h.

After incubation, 30 μL of an aqueous resazurin (Sigma) solution (0.02%) was added to the microplates to observe bacterial growth. Development of a blue and pink color indicated absence and presence of bacterial growth, respectively. To determine the MBC, an aliquot of the inoculum was removed from each well prior to addition of resazurin (Sigma) and seeded in an appropriate culture medium.

### Time-kill curves

Time-kill assays against the anaerobic strains *P. gingivalis* (ATCC 33277) and *P. micros* (clinical isolate) and the microaerophilic strains *S. mutans* (ATCC 25275), *S. sobrinus* (ATCC 33478), and *L. casei* (ATCC 11578 and clinical isolate) were performed in triplicate, as described by D'arrigo et al. (2010). All the results are expressed as the mean ± S.E.M. Tubes containing the most promising metabolites at final concentrations equal to the MBC values for the respective strains were inoculated with the target microorganism at an initial bacterial density of 5 × 10^5^ CFU mL^−1^ for the anaerobic facultative strains and 5 × 10^6^ CFU mL^−1^ for the anaerobic strains, followed by anaerobic or microaerophilic incubation conditions. To count viable colonies, aliquots were removed at 0 min and 30 min and at 6, 12, 18, and 24 h for microaerophilic bacteria, and at 0 min and 30 min and at 6, 12, 18, 24, 48, and 72 h for anaerobic bacteria. The diluted samples (50 μL) were spread onto appropriate agar, incubated at 37°C under appropriate atmosphere, and counted after the growth period. Time-kill curves were constructed by plotting log_10_ CFU mL^−1^ vs. time on the Graphpad Prism (version 5.0) software. Promising metabolites at their MBC and a suspension of bacteria without the added metabolites were used as the positive and the negative control, respectively.

### Antibiofilm activity evaluation

The Minimum Inhibitory Concentration of Biofilm (MICB_50_) of the most promising metabolites against the bacteria evaluated in this study was determined on the basis of the minimum concentration of antimicrobial agent that was able to inhibit biofilm formation by at least 50% (Wei et al., 2006). For this purpose, a microdilution plate assay was used according to the CLSI guidelines (CLSI, 2007, 2009), with some modifications. This method was similar to the MIC assay conducted for planktonic cells except that the inoculum was adjusted at a higher concentration so that it could adhere to the microplate to form the biofilm. Two-fold serial dilutions of each sample were prepared in the wells of a 96-well polystyrene tissue culture plate (TPP, Trasadingen, Switzerland) containing appropriate medium at a volume of 200 μL per well. The final concentrations of the most promising metabolites ranged from 0.195 to 400 μg mL^−1^. Chlorhexidine dichlorohydrate (Sigma) at a concentration between 0.115 and 59 μg mL^−1^ was assessed as negative control; the bacterial strains in the absence of the antibacterial agent were used as positive controls, and the inoculum was adjusted to give a cell concentration of 1 × 10^6^ CFU mL^−1^ for all the bacteria. *P. gingivalis* (ATCC 33277) and *P. micros* (clinical isolate) were incubated in an anaerobic chamber, and the microaerophilic strains *S. mutans* (ATCC 25275), *S. sobrinus* (ATCC 33478), and *L. casei* (ATCC 11578 and clinical isolate) were incubated in a microaerophilic jar system. Biofilm formation was quantified, and the number of microorganisms was counted by using the methodology described by da Silva et al. (2014), with some modifications.

### Synergistic antimicrobial activity

Checkerboard assays were performed according to the protocol previously described by White et al. (1996) to investigate the *in vitro* antimicrobial efficacy of the combination of the oleoresin or (-)-polyalthic acid with chlorhexidine (Sigma). The synergy tests were carried out in triplicate, and concentrations of each compound were combined by using a standard MIC format against 5 × 10^5^ CFU mL^−1^ of the microaerophilic strain and 5 × 10^6^ CFU mL^−1^ of the anaerobic strain. To evaluate the synergistic effect of the most promising metabolites and chlorhexidine, the fractional inhibitory concentration (FIC) index values were calculated on the basis of the equation previously established in the literature (White et al., 1996). Synergy was defined as FIC ≤ 0.5, and additivity was defined as FIC > 0.5 but <1. Indifference was defined as FIC ≥ 1 but <4, whereas antagonism was defined as FIC ≥ 4 (Lewis et al., 2002).

### Cytotoxicity assay

Chinese hamster lung fibroblasts (V79) were employed in this study. The cell line was maintained as monolayers in a plastic culture flask (25 cm^2^) in a culture medium (HAM-F10 + DMEM, 1:1, or only DMEM, Sigma) supplemented with 10% fetal bovine serum (Nutricell, Campinas, SP, Brazil), antibiotics (streptomycin 0.01 mg mL^−1^ and penicillin 0.005 mg mL^−1^; Sigma-Aldrich), and Hepes 2.38 μg mL^−1^ (Sigma), at 37°C, with 5% CO_2_ or in a BOD-type chamber.

Cytotoxicity was measured by using the *in vitro* Toxicology Colorimetric Assay Kit (XTT; Roche Diagnostics, Indianapolis, Indiana, EUA) according to the manufacturer's instructions. For these experiments, 1 × 10^4^ cells were plated onto 96-well microplates. Each well received 100 μL of HAM-F10/DMEM or DMEM containing the *C. duckei* oleoresin, polyalthic acid, or eperu-8(20)-15,18-dioic acid at concentrations ranging from 2.43 to 5,000 μg mL^−1^. The negative (without treatment), solvent (Tween 80 0.25%), and positive (doxorubicin, DXR, Zodiac, Pindamonhangaba, SP, Brazil) controls were included. After incubation at 37°C for 24 h, the medium was removed; the cells were washed twice with 100 μL of phosphate buffered saline (PBS) and exposed to 100 μL of HAM-F10 medium without phenol red. Then, 50 μL of XTT was added to each well. The microplates were covered and incubated at 37°C for 17 h. The absorbance of the samples was determined by using a multiplate reader (ELISA, Tecan—SW Magellan vs. 5.03 STD 2PC) at a test wavelength of 492 nm and a reference wavelength of 690 nm (Roehn et al., 1991). The experiments were conducted in triplicate, and the antiproliferative activity was assessed by using the parameter of 50% inhibition of cell growth (IC_50_) with the aid of GraphPad Prism 5.0.

## Results and discussion

**Figure 1:**
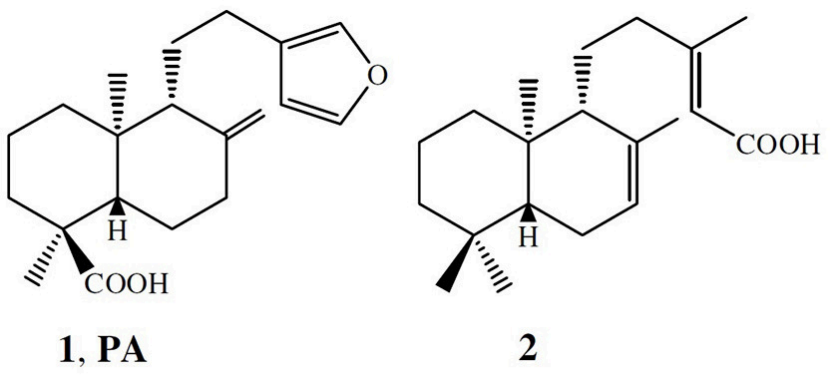
Chemical structures of the main terpenes isolated from the *C. duckei* oleoresin.

Figure 1 illustrates the chemical structures of the secondary compounds obtained from *C. duckei* and evaluated herein. According to Rios and Recio (2005) and Gibbons (2008), a promising plant extract must have MIC lower than 100 μg mL^−1^, whilst pure compounds must display MIC values lower than 10 μg mL^−1^. Polyalthic acid gave MIC values ranging between 12.5 and 100 μg mL^−1^ for the cariogenic strains. Table 1 summarizes the MIC and MBC values for the assessed bacteria involved in endodontic infections and dental caries. The oleoresin displayed good results for all the cariogenic strains and for nine representative strains of endodontic infections with MIC values until 100 μg mL^−1^. The MBC assay showed that the oleoresin exerted bactericidal effects on all the cariogenic strains except *L. casei* (clinical isolate), for which we detected bacteriostatic action. The oleoresin exhibited bactericidal activity against three strains representing endodontic infections, namely the *P. gingivalis, F. nucleatum*, and *P. micros* clinical isolates. (–)-Polyalthic acid presented MIC values varying between 12.5 and 100 μg mL^−1^ for the cariogenic strains. Regarding MCB, (–)-polyalthic acid did not show bactericidal effect against three strains only. For the strains representing endodontic infections, polyalthic acid afforded MIC values lying between 6.25 and >400 μg mL^−1^ with bacteriostatic results for four strains. Eperu-8(20)-15,18-dioic acid was not effective against any of the strains tested here: MIC and MBC values ranged between 50 and >400 μg mL^−1^.

**Table 1:** Antibacterial potential of the *Copaifera duckei* Dwyer oleoresin, (–)-polyalthic acid, and eperu-8(20)-15,18-dioic acid against bacteria involved in dental caries and endodontic infections.

**Microorganisms**		**Minimum Inhibitory Concentration (MIC)/Minimum Bactericidal Concentration (MBC) −μg mL^−1^**				
		**Oleoresin**	**(–)-Polyalthic acid**	**Eperu-8(20)-15,18-dióic acid**	**Chlorhexidine**	**Metronidazole**
Cariogenic strains	*S. sobrinus* (ATCC 33478)	25/25	25/25	^*^	0.92/0.92	–
	*S. mitis* (ATCC 49456)	25/25	25/25	^*^	3.68/3.68	–
	*S. mutans* (ATCC 25175)	25/25	25/25	400/400	0.92/0.92	–
	*S. salivarius (*ATCC 25975)	25/25	50 /50	^*^	0.92/0.92	–
	*S. salivarius* (clinical isolate)	25/25	25/50	^*^	0.92/0.92	–
	*S. sanguinis (*ATCC 10556)	25/25	25/50	^*^	7.37/7.37	–
	*S. sanguinis* (clinical isolate)	25/25	50/50	400/400	3.68/3.68	–
	*L. casei* (ATCC 11578)	25/25	50/50	400/400	3.68/3.68	–
	*L. casei* (clinical isolate)	25/50	12,5/12,5	^*^	3.68/3.68	–
	*E. faecalis* (ATCC 4082)	50/50	25 /25	^*^	7.37/7.37	–
	*E. faecalis* (clinical isolate)	50/50	100/200	^*^	7.37/7.37	–
Endodontic infection strains	*A. actinomycetemcomitans* (ATCC 43717)	12.5/25	25/25	^*^	–	–
	*P. gingivalis* (ATCC 33277)	6.25/6.25	6.25/6.25	50/100	1.84/1.84	–
	*P. gingivalis* (clinical isolate)	100/200	50/100	400/>400	–	–
	*P. intermedia* (clinical isolate)	^*^	100/100	400/400	–	–
	*F. nucleatum* (ATCC 25586)	50/50	25/25	200/400	–	–
	*F. nucleatum* (clinical isolate)	50/100	50/100	^*^	–	–
	*A. naeslundii* (ATCC 19039)	12.5/12.5	25/25	400/>400	7.37/7.37	–
	*A. naeslundii* (clinical isolate)	400/400	200/400	^*^	–	–
	*A. viscosus* (clinical isolate)	^*^	400/>400	^*^	–	–
	*P. nigrescens* (ATCC 33563)	50/50	50/100	400/>400	–	–
	*P. buccae* (clinical isolate)	^*^	100/200	400/400	–	–
	*P. micros* (clinical isolate)	25/25	6.25/12.5	50/100	7.37/7.37	–
	*B. fragilis* (ATCC 25285)	25/25	50/50	^*^	–	1.47/1.47
	*B. thetaiotaomicron* (ATCC 29741)	–	–	–	–	2.95/2.95

* *Inactive at the evaluated concentration (MIC values higher than 400 mg L^−1^). –, Not evaluated*.

Santos et al. (2013) investigated the antibacterial activity of the *C. duckei* oleoresin against bacterial of clinical and food interest, namely *Staphylococcus aureus* (ATCC 29213, 25923 and 33591), *E. faecalis* (ATCC 29212), *Listeria monocytogenes* (ATCC 15313), *B. cereus* (ATCC 11778), *Salmonella* Typhimurium (ATCC 14028), *Escherichia coli* (ATCC 25922), *Pseudomonas aeruginosa* (ATCC 27853), and *Staphylococcus epidermidis* and *Shigella sonnei*, which were both clinical isolates. The oleoresin was active against nine of the 11 tested bacterial strains. *B. cereus* was the most sensitive: the oleoresin MIC was 31.25 μg mL^−1^, which denoted bactericidal action. The authors verified that the *C. duckei* oleoresin is a potential antibacterial agent and suggested that this oil can be used as a therapeutic alternative, mainly against *B. cereus* (ATCC 25922). Here, the oleoresin gave MIC values of 25 μg mL^−1^ against most cariogenic strains, and it was the most promising against *P. gingivalis* (ATCC 33277), *P. micros* (clinical isolate), and *A. naeslundii* (ATCC 19039), with MIC values of 6.25, 25, and 12.5 μg mL^−1^, respectively. These results attested to the antibacterial potential of the *C. duckei* oleoresin.

Moraes et al. (2016) studied the antibacterial activity of the *C. oblongifolia* oleoresin against bacteria involved in caries and endodontic infections, to achieve promising MIC and MBC values spanning from 25 to 200 μg mL^−1^ as well as encouraging MIC values against *S. sanguinis* (ATCC 10556 and clinical isolate), *S. mutans* (ATCC 25175), *S. mitis* (ATCC 49456), *L. casei* (ATCC 11578 and clinical isolate strains), *P. gingivalis* (ATCC 33277), *P. micros* (clinical isolate), and *A. actinomycetemcomitans* (ATCC 43717). In our study, the *C. duckei* oleoresin displayed good results against the same bacteria evaluated by Moraes et al. (2016), with MIC values ranging from 6.25 to 50 μg mL^−1^, which constituted a bactericidal effect. The exception was *A. actinomycetemcomitans*, against which the oleoresin was bacteriostatic.

Bardají et al. (2016) assessed the *Copaifera reticulata* oleoresin against the causative agents of tooth decay and periodontitis, to obtain the best result against *P. gingivalis* (ATCC 33277), with MIC value of 6.25 μg mL^−1^. In the present work, the *C. duckei* oleoresin provided the same result against *P. gingivalis* (ATCC 33277), which corresponded to bactericidal action. Polyalthic acid also afforded good results for both groups of bacteria tested herein, with MIC values lower than 10 μg mL^−1^ for the anaerobic strains *P. gingivalis* (ATCC 33277) and *P. micros* (clinical isolate).

There are no reports on the use of pure compounds of the *C. duckei* oleoresin against bacteria. However, our research group has already obtained pure substances from *C. langsdorffii* and found good results for copalic acid against cariogenic bacteria (Souza et al., 2011a) and periodontal anaerobic bacteria (Souza et al., 2011b).

Based on our promising MIC results, we examined the bacterial death kinetics (time-kill assays), the *in vitro* antibiofilm activity (MICB_50_), and the synergistic effect of the *C. duckei* oleoresin and polyalthic acid associated with chlorhexidine.

**Figure 2:**
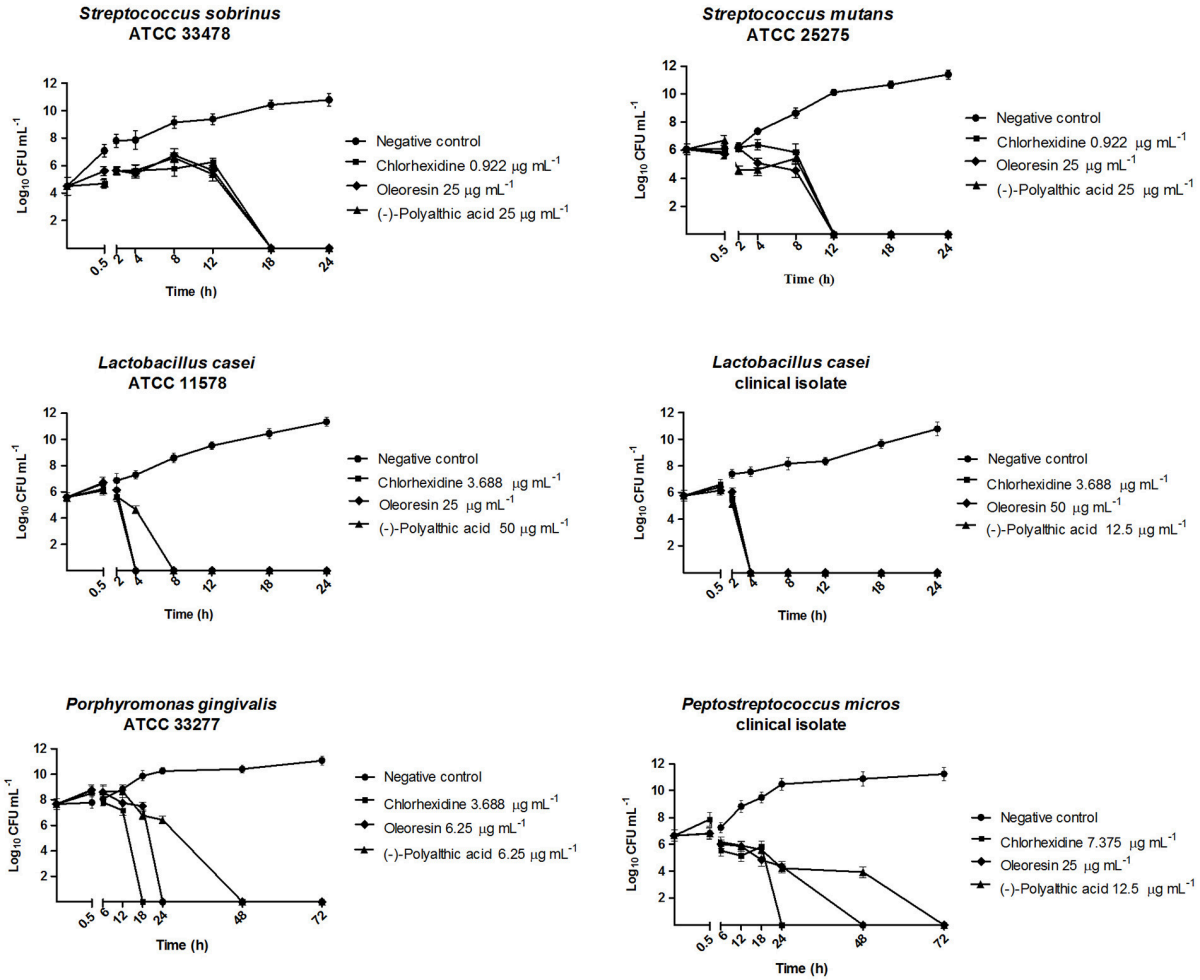
Bactericidal kinetics of the *Copaifera duckei* Dwyer oleoresin and (–)-polyalthic acid against bacteria involved in dental caries and endodontic infections.

We accomplished the time-kill curve assay (Figure 2) against two anaerobic strains and four microaerophilic strains, which best represented endodontic and cariogenic infections and provided the greatest results in the MIC and MBC assays. In this assay, the bactericidal effect of the oleoresin and polyalthic acid varied. We highlight the results obtained against *L. casei* (ATCC 11578 and clinical isolate), which had inferior bactericidal effect after incubation for 8 h. According to Petersen et al. (2004), bactericidal activity corresponds to a reduction of >3 log10 CFU mL^−1^ in the original inoculum, whereas bacteriostatic activity refers to maintenance of the original inoculum concentration or reduction of <3 log_10_ CFU mL^−1^ in the original inoculum.

Santos et al. (2013) reported the time-kill assay of the *C. duckei* oleoresin at 15.62, 31.25, 62.5, and 125 μg mL^−1^ against *B. cereus* (ATCC 25922). The oleoresin exerted bactericidal effect on *B. cereus* in <4 h, at concentrations ranging from 31.25 to 125 μg mL^−1^ (1–4 times the MIC value). We also achieved similar results with the oleoresin against *L. casei* (ATCC 11578 and clinical isolate) and with polyalthic acid against *L. casei* (clinical isolate), which afforded bactericidal action after incubation for 4 h. Souza et al. (2011a) tested *C. langsdorffii* copalic acid against *S. mutans* (ATCC 25275), to find that copalic acid only inhibited inoculum growth during the first 12 h. The authors concluded that copalic acid displayed a bacteriostatic effect during this time, but its bactericidal action was clearly noted thereafter (between 12 and 24 h).

Souza et al. (2011b) also investigated *C. langsdorffii* copalic acid against *P. gingivalis* (ATCC 33277) in a time-kill curve assay in which this compound was tested at 3.1, 6.2, and 12.4 μg mL^−1^ (one, two, and three times its MBC, respectively); chlorhexidine at its MBC value (0.9 μg mL^−1^) was the positive control. Copalic acid 3.1 μg mL^−1^ completely killed *P. gingivalis* after incubation for only 24 h. However, the data suggested that copalic acid only inhibited inoculum growth during the first 12 h. Therefore, copalic acid displayed a bacteriostatic effect during this time, but its bactericidal action was clearly noted thereafter (between 12 and 24 h). In our study, *P. gingivalis* (ATCC 33277) behaved similarly. It was killed within 24 and 48 h of exposure to the *C. duckei* oleoresin and to polyalthic acid, respectively. Leandro et al. (2016) conducted a time-kill assay of the hydroalcoholic extract from *C. trapezifolia* leaves at 100 μg mL^−1^ against *P. gingivalis* (ATCC 33277) and *P. micros* (clinical isolate) and detected bactericidal activity within 72 h.

Moraes et al. (2016) accomplished a time-kill assay for the *C. oblongifolia* oleoresin at 100 mg mL^−1^, to find that this oleoresin exerted a bactericidal effect against *L. casei* (ATCC) and *A. actinomycetemcomitans* within 24 h. In addition, these authors tested the same *C. oblongifolia* oleoresin at 25 mg mL^−1^ against *P. micros* (clinical isolate), to verify that the number of microorganisms decreased by over 3 log_10_ CFU mL^−1^ after 48 h, and that bactericidal activity emerged at 72 h of incubation. In the present study, both the *C. duckei* oleoresin and polyalthic acid reduced the number of microorganisms by at least 3 log_10_ CFU mL^−1^ at 48 h of incubation for all the evaluated anaerobic strains.

In a time-kill assay employing the *C. reticulata* oleoresin at concentrations between 50 and 100 mg mL^−1^, Bardají et al. (2016) found bactericidal activity against *F. nucleatum* (ATCC 25586) and *S. mitis* (ATCC 49456) after 4 h, against *P. nigrescens* (ATCC 33563) after 6 h, against *P. gingivalis* (ATCC 33277) and *L. casei* (clinical isolate) after 12 h, and against *S. salivarius* (ATCC 25975) and *S. mutans* (ATCC 25175) after 18 h. Here, *L. casei* (ATCC 11578) exposed to polyalthic acid 25 μg mL^−1^ was killed within 4 h of incubation. We achieved similar results for *L. casei* (clinical isolate) exposed to the *C. duckei* oleoresin and (–)-polyalthic acid (Figure 2).

**Figure 3:**
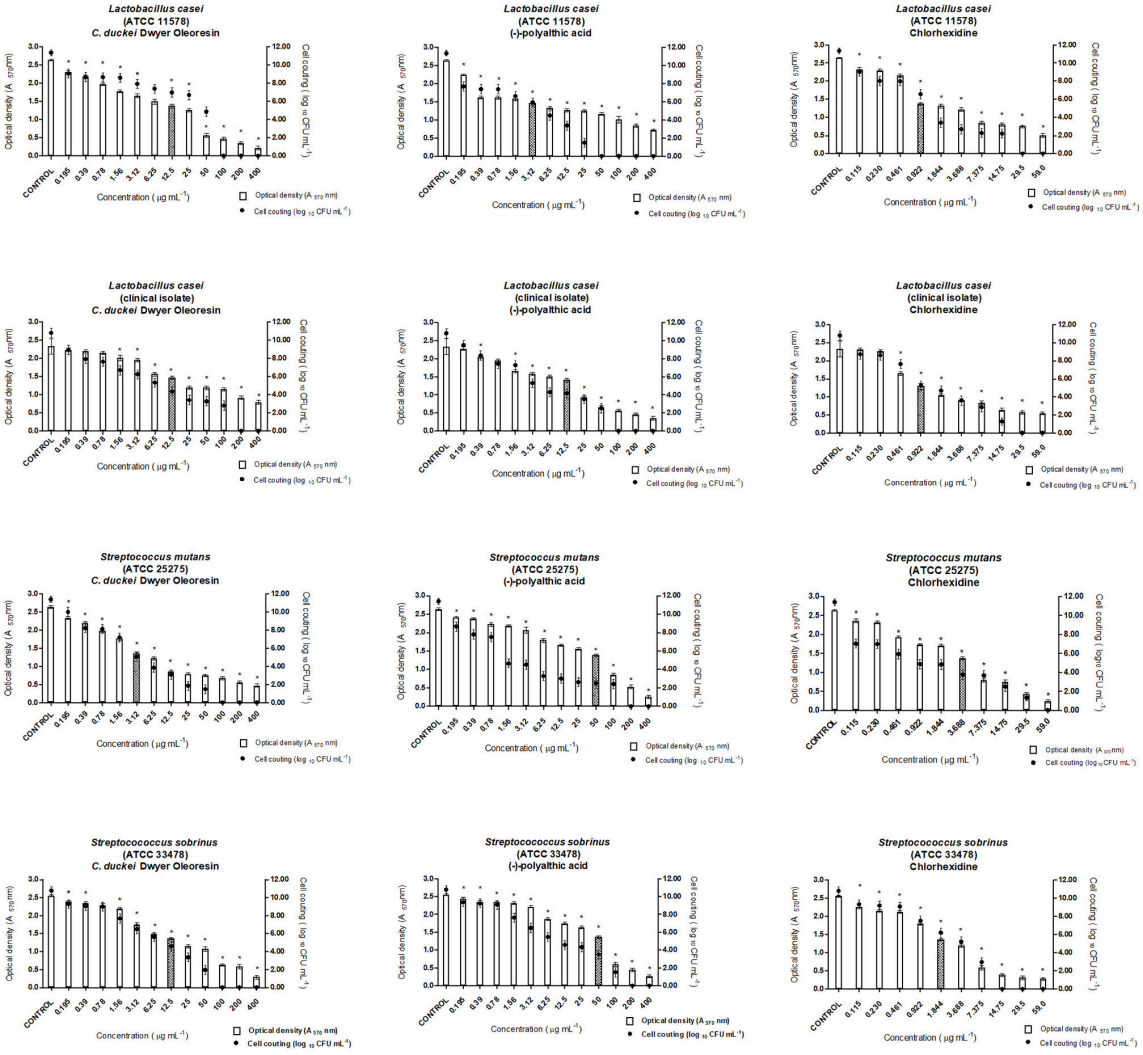
Antibiofilm activity of the *Copaifera duckei* Dwyer oleoresin and (–)-polyalthic acid as demonstrated by optical density (A_570_) and number of microorganisms (Log_10_ CFU mL^−1^) against cariogenic bacteria. The experiments were performed in triplicate and statistical significance was examined by Student's *t*-test. Results are indicated as means ± SDs. *Significantly different from the negative control group (*P* < 0.05). Filled bars correspond to MICB_50_ concentration.

**Figure 4:**
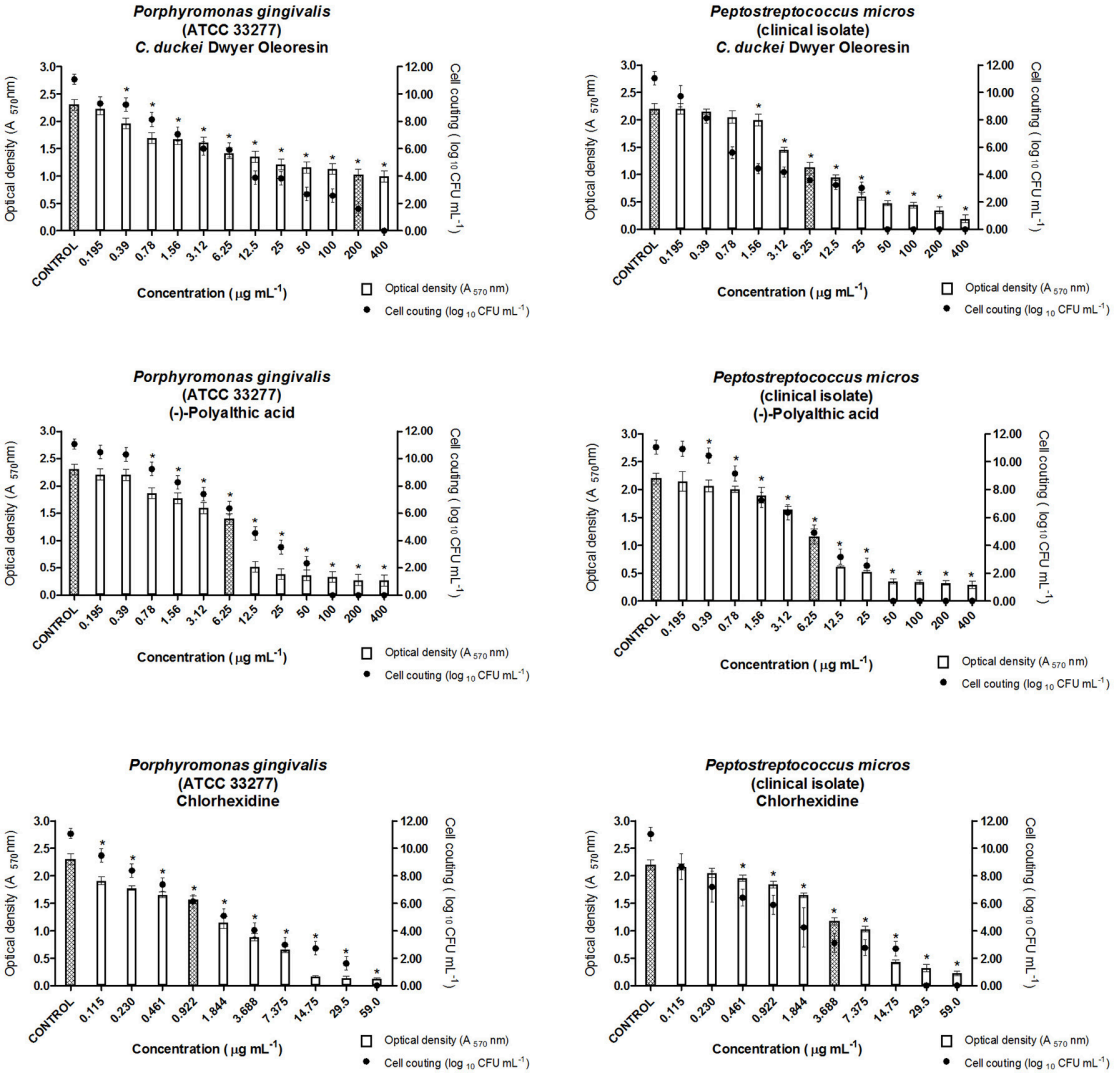
Antibiofilm activity of oleoresin and (–)-polyalthic acid as demonstrated by optical density (A_570_) and number of microorganisms (Log_10_ CFU mL^−1^) against bacteria that cause endodontic infections. The experiments were performed in triplicate and statistical significance was examined by Student's *t*-test. Results are indicated as means ± SDs. *Significantly different from the negative control group (*P* < 0.05). Filled bars correspond to MICB_50_ concentration.

According to Stewart and Costerton (2001), biofilms are more resistant to antimicrobial agents as compared to planktonic cells. During MICB_50_ evaluation, the oleoresin and polyalthic acid displayed promising results against all the tested bacteria. We highlight the results found for *L. casei* (ATCC 11578) exposed to polyalthic acid, which displayed MICB_50_ of 3.12 μg mL^−1^ (Figures 3, 4). Fux et al. (2003) affirmed that the concentration of a drug required to eliminate sessile bacteria can vary from 10- to 1,000-fold when it comes to eliminating planktonic bacteria. Most of the evaluated strains showed MICB_50_ values lower than the MIC values. However, cell counting demonstrated that at all the concentrations that represented MICB_50_, there still were living cells. According to Wei et al. (2006), avoiding biofilm formation is more important than destroying the fully developed biofilm. Spectrophotometric readings (O.D.) and microorganism count (log_10_ CFU mL^−1^) can show the ability of antimicrobial agents to inhibit biofilm formation (antibiofilm activity). The existing methods have limitations such as long processing time, incompatibility with screening techniques, expensive reagents, and measurement of mass instead of cell viability. Despite these limitations, the combination of both techniques provides reliable results concerning biofilm activity (Kharazmi et al., 1999; Polonio et al., 2001; Walters et al., 2003; da Silva et al., 2014).

According to our study, the *C. duckei* oleoresin at concentrations of 200 and 6.25 μg mL^−1^ inhibited at least 50% of biofilm formation in the case of *P. gingivalis* (ATCC 33277) and *P. micros* (clinical isolate), respectively (Figure 4). The pure compound (–)-polyalthic acid at a concentration of 6.25 μg mL^−1^ inhibited at least 50% of biofilm formation of *P. gingivalis* (ATCC 33277) and *P. micros* (clinical isolate).

Moraes et al. (2016) investigated the ability of the *C. oblongifolia* oleoresin to inhibit biofilm formation. They found MICB_50_ of 400 μg mL^−1^ for *L. casei* and *P. micros*, 200 μg mL^−1^ for *S. mutans* and *A. actinomycetemcomitans*, and 100 μg mL^−1^ for *S. mitis* and *P. gingivalis*. Bardají et al. (2016) evaluated the MICB_50_ of the *C. reticulata* oleoresin. At 50, 100, and 200 μg mL^−1^, this oleoresin inhibited biofilm formation by at least 50% in the case of *L. casei*, and *S. salivarius*, and *S. mitis*, respectively. Compared to the results of Bardají et al. (2016), in this work inhibition of biofilm formation by cariogenic strains provided by the *C. duckei* oleoresin and (–)-polyalthic acid was more promising: from 3.12 to 12.5 μg mL^−1^ and from 12.5 to 50.0 μg mL^−1^, respectively (Figure 3).

We also evaluated the synergistic effect of chlorhexidine and the *C. duckei* oleoresin or polyalthic acid against some of the assayed bacteria (Table 2). The checkerboard methodology described by Lewis et al. (2002) did not reveal any synergistic effects for the tested combinations. The FICI results only evidenced additive and indifferent interactions. Bardají et al. (2016) studied the combination of chlorhexidine with the *C. reticulata* oleoresin, to find an additive effect for *S. mutans* (ATCC 25175) and *S. mitis* (ATCC 49456). Moraes et al. (2016) also detected an additive effect for the combination of chlorhexidine with the *C. oblongifolia* oleoresin against *S. mitis* (ATCC 49456) and *A. actinomycetemcomitans* (ATCC 43717). In turn, Leandro et al. (2016) did not verify any synergistic effect for the combination of the hydroalcoholic extract from *C. trapezifolia* leaves with chlorhexidine. These results corroborate with our present findings.

**Table 2:** FIC indexes of the combined action of the *Copaifera duckei* Dwyer oleoresin or (–)-polyalthic acid and chlorhexidine against bacteria involved in dental carie and endodontic infections.

**Microorgranisms**		**MIC 1 (μg mL^−1^)**	**MIC 2 (μg mL^−1^)**	**FIC 1 (μg mL^−1^)**	**FIC 2 (μg mL^−1^)**	**FIC index**	**Interpretation**
Oleoresin × CDH	*S. mutans* (25175 ATCC)	50	0.922	25	0.230	0.74	Additivity
	*S. sobrinus* (33478 ATCC)	50	0.922	50	0.461	1.50	Indifference
	*L. casei* (11578 ATCC)	25	3.688	25	0.461	1.12	Indifference
	*L. casei* (Clinical isolate)	25	3.688	12.5	0.461	0.62	Additivity
	*P. gingivalis* (33277 ATCC)	6.25	1.844	12.5	1.844	3.00	Indifference
	*P. micros* (Clinical isolate)	25.0	7.375	6.25	14.75	2.25	Indifference
Polyalthic acid × CDH	*S. mutans* (25175 ATCC)	50	0.922	50	0.230	1.24	Indifference
	*S. sobrinus* (33478 ATCC)	12.5	0.922	12.5	0.922	2.00	Indifference
	*L. casei* (11578 ATCC)	25	7.375	25	0.922	1.25	Indifference
	*L. casei* (Clinical isolate)	25	7.375	25	0.461	1.12	Indifference
	*P. gingivalis* (33277 ATCC)	3.12	1.844	3.12	3.688	3.00	Indifference
	*P. micros* (Clinical isolate)	12.5	7.375	6.25	14.75	2.5	Indifference

*1. Oleoresin or (–)-polyalthic acid; 2. Chlorhexidine*.

**Figure 5:**
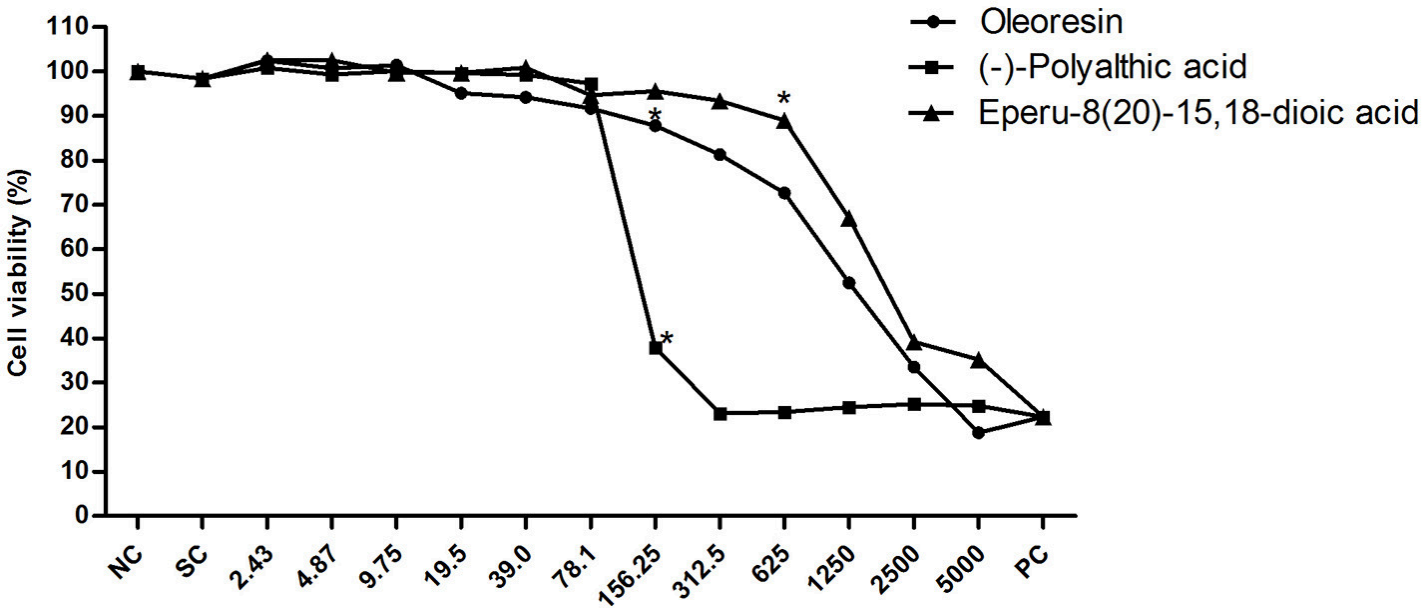
Cell viability of the V79 cell line when exposed to different concentrations of the *Copaifera duckei* Dwyer oleoresin, (–)-polyalthic acid, and eperu-8(20)-15,18-dioic acid as assessed by the XTT colorimetric method. The values are the mean and standard deviation. *Significantly different from the negative control group (*P* < 0.05). IC_50_ values were 777.4 ± 8.3; 127.93 ± 10.97, and 1441.33 ± 13.43 for oleoresin, (–)-polyalthic acid and eperu-8(20)-15,18-dioic acid, respectively.

Finally, we investigated the cytotoxic potential of the *C. duckei* oleoresin, polyalthic acid, and eperu-8(20)-15,18-dioic acid (Figure 5). The oleoresin and polyalthic acid afforded IC_50_ values of 777.4 ± 8.3 and 127.3 ± 10.97 μg mL^−1^, respectively. Eperu-8(20)-15,18-dioic acid showed IC_50_ values of 1441.33 ± 13.43 μg mL^−1^. In conclusion, eperu-8(20)-15,18-dioic acid was not cytotoxic to the V79 cell line, and it did not display antibacterial activity at MIC and MBC. The oleoresin and polyalthic acid did not present cytotoxicity at the MIC and MBC concentrations. These results suggested that these natural products could be safely applied to treat oral diseases. Leandro et al. (2016) also evaluated the cytotoxicity of the hydroalcoholic extract from *C. trapezifolia* leaves against the V79 cell line and found cytotoxicity at concentrations above 156 μg mL^−1^. As reported by Moraes et al. (2016), the *C. oblongifolia* oleoresin was cytotoxic activity against the V79 cell line at concentrations ≥625 μg mL^−1^. Bardají et al. (2016) treated GM07492-A cells with the *C. reticulata* oleoresin, to demonstrate that concentrations up to 39 μg mL^−1^ significantly reduced cell viability as compared to the negative control; IC_50_ was equal to 51.85 ± 5.4 μg mL^−1^.

## Conclusions

The *C. duckei* oleoresin and polyalthic acid are important substances in the search for new antibacterial agents against the tested pathogens involved in dental caries and endodontic infections.

## Author contributions

CM, RV, SA, and JB: Conceived the idea for this study; FA, CM, and JB: Participated in the study design; FA, JA, and GA: Conducted the antibacterial assays; PO and DT: Conducted the cytotoxicity assay; FA and CM: Organized the data and evaluated their quality; CM, RV, SA, and JB: Critically reviewed the manuscript. All authors have read and approved the final manuscript.

### Conflict of interest statement

The authors declare that the research was conducted in the absence of any commercial or financial relationships that could be construed as a potential conflict of interest.

## Abbreviations

ATCC: American Type Culture Collection
CDH: Chlorhexidine Dihydrochloride
CFU: Colony Forming Units
CLSI: Clinical and Laboratory Standards Institute
DMSO: Dimethyl Sulfoxide
FIC: Fractional Inhibitory Concentration
MBC: Minimum Bactericidal Concentration
MIC: Minimum Inhibitory Concentration
MICB_50_: Minimum Bactericidal Concentration
TSA: Tryptic Soy Agar
TSB: Tryptic Soy Broth.
